# Coronary Computed Tomography Angiography Based Assessment of Endothelial Shear Stress and Its Association with Atherosclerotic Plaque Distribution *In-Vivo*

**DOI:** 10.1371/journal.pone.0115408

**Published:** 2015-01-30

**Authors:** Holger Hetterich, Ahmad Jaber, Moritz Gehring, Adrian Curta, Fabian Bamberg, Nenad Filipovic, Johannes Rieber

**Affiliations:** 1 Institute of Clinical Radiology, Ludwig-Maximilians-University Hospital, Munich, Germany; 2 Department of Cardiology, Ludwig-Maximilians-University Hospital, Munich, Germany; 3 Faculty of Mechanical Engineering, University of Kragujevac, Kragujevac, Serbia; 4 Department of Cardiology and Intensive Care Medicine, Heart Center Munich-Bogenhausen, Munich, Germany; Medical University Innsbruck, AUSTRIA

## Abstract

**Purpose:**

The relationship between low endothelial shear stress (ESS) and coronary atherosclerosis is well established. ESS assessment so far depended on invasive procedures. The aim of this study was to demonstrate the relationship between ESS and coronary atherosclerosis by using non-invasive coronary computed tomography angiography (CTA) for computational fluid dynamics (CFD) simulations.

**Methods:**

A total number of 7 consecutive patients with suspected coronary artery disease who received CTA and invasive angiography with IVUS analysis were included in this study. CTA examinations were performed using a dual-source scanner. These datasets were used to build a 3D mesh model. CFD calculations were performed using a validated CFD solver. The presence of plaque was assumed if the thickness of the intima-media complex exceeded 0.3 mm in IVUS. Plaque composition was derived by IVUS radiofrequency data analysis.

**Results:**

Plaque was present in 32.1% of all analyzed cross-sections. Plaque prevalence was highest in areas of low ESS (49.6%) and high ESS (34.8%). In parts exposed to intermediate-low and intermediate-high ESS few plaques were found (20.0% and 24.0%) (p<0.001). Wall thickness was closely associated with local ESS. Intima-media thickness was 0.43±0.34mm in low and 0.38±0.32mm in high ESS segments. It was significantly lower when the arterial wall was exposed to intermediate ESS (0.25±0.18mm and 0.28 ± 0.20mm) (p<0.001). Fibrofatty tissue was predominately found in areas exposed to low ESS (p≤0.023).

**Conclusions:**

In this study a close association of atherosclerotic plaque distribution and ESS pattern could be demonstrated in-vivo. Adding CFD analysis to coronary CTA offers the possibility to gather morphologic and physiologic data within one non-invasive examination.

## Introduction

The localization of coronary atherosclerotic lesions is not randomly distributed but shows a characteristic pattern with preferred locations at branches or bends [1]. The complex geometry of the vessel causes a distortion of the laminar flow up to local flow stagnation or reversal. This results in locally different levels of endothelial shear stress (ESS). Especially low ESS has been linked with the initiation and progression of atherosclerosis [2–4].

Shear stress is difficult to measure directly in-vivo. Using computational fluid dynamics (CFD) ESS and other flow dependent parameters can be calculated if an accurate description of the vessel geometry is available. In the coronary circulation most CFD studies so far relied on invasive procedures such as invasive angiography and intravascular ultrasound (IVUS), limiting the results to high risk patients with indication for cardiac catheterization [5–7].

Besides these invasive techniques, 3-dimensional (3D) models can today be obtained by non-invasive coronary computed tomography angiography (CTA). Several studies showed that CTA models are accurate and can be used for CFD calculations [8–10]. However, so far ESS pattern calculated based on CTA models has not been linked to atherosclerotic plaque distribution in patients in-vivo.

The aim of this study was to calculate ESS based on the vessel geometry as obtained by CTA and compare these findings with the distribution and composition of coronary atherosclerotic plaques as assessed by IVUS and radiofrequency data analysis (RF) in patients without priory known coronary artery disease.

## Methods

### Study design and overview

This project was designed as a prospective in-vivo feasibility study. The protocol was approved by the ethics committee of the Ludwig-Maximilians University and written informed consent was obtained from all patients before entering the study. The study complies with the declaration of Helsinki of 1975, as revised in 2008. Consecutive patients underwent CTA and IVUS evaluation. CTA models were used for ESS calculations, IVUS data for plaque assessment only. Both were matched using multiple anatomical landmarks and the association of ESS level and plaque distribution was assessed.

### Patients

Patients underwent coronary CTA if they had symptoms suggestive for coronary artery disease with a low to intermediate likelihood and stress testing was not possible or the results of stress testing were not conclusive or uninterpretable [11]. Patients with acute chest pain, recent acute coronary syndromes, known coronary artery disease, impaired renal function (glomerular filtration rate <60 ml/min) allergy to contrast media, active cancer or a live expectancy of less than one year were not included in this study. If significant coronary artery disease could not be excluded by CTA, patients received coronary angiography and IVUS evaluation. Vessels with significant stenosis of more than 30% by quantitative coronary angiography were not included in the analysis.

### Coronary CTA

The CTA examinations were performed on a dual source CT scanner (Somatom Definition, Siemens AG, Healthcare Sector, Forchheim, Germany) with the following parameters: gantry rotation time 0.33 s, temporal resolution 83 msec, pitch adapted to heart rate (0.2–0.43), tube voltage 120 kV, tube current 560 mA with electrocardiogram (ECG) triggered tube current modulation. All patients received 0.8 mg nitroglycerine sublingually 2 minutes before the scan. The contrast agent (Ultravist 370, Schering AG, Berlin, Germany) was continuously injected in the right or left antecubital vein at a volume and flow rate adapted to the patient’s body weight followed by a flush of 50 ml normal saline solution using an 18 gauge venous catheter. In a region of interest in the ascending aorta bolus tracking was performed and data acquisition was automatically started 4 sec after attenuation reached 100 Hounsfield units. CT data sets were retrospectively reconstructed at 20%, 40% and 80% of the R-R interval obtained by ECG with a slice thickness of 0.75 mm and an increment of 0.5 mm using a medium soft-tissue convolution kernel (B26f). The phase with the best image quality and the least motion artifacts was used for further processing.

### Invasive coronary angiography and IVUS

The ostia of the coronary arteries were cannulated via femoral approach using standard Judkins technique. To achieve maximum dilatation of the coronary arteries 0.25 mg of nitroglycerin were injected intracoronarily. Angiographic images from different standardized angles were obtained to achieve an optimal visualization of the vessel without shortening or overlapping.

Subsequently a guidewire was introduced into the vessel and the IVUS probe was advanced. The studies were performed using a commercially available imaging system (S5i, Volcano Corp., Rancho Cordova, CA, USA), which allows ECG gated data acquisition. The ultrasound probe (Eagle Eye Gold, Volcano Corp., Rancho Cordova, CA, USA) had a diameter of 2.9 F. Using a carrier frequency of 20 MHz a penetration depth of about 8 mm can be achieved. The axial resolution is 80 μm, the lateral resolution 200 μm. Data for both grayscale IVUS and RF data were simultaneously acquired during a standardized motorized pullback at a speed of 0.5 mm/sec.

### Data processing

#### Geometric reconstruction

**Figure 1:**
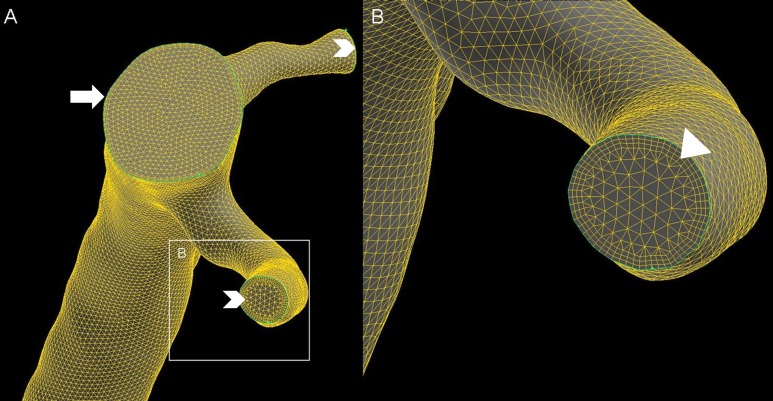
Three dimensional mesh model of a right coronary artery. Panel (A) shows the inflow (arrow) at the ostium of a right coronary artery and outflows of two small side branches (chevrons). Panel (B) shows a magnification of the outflow shown in panel (A). Notice the three boundary layers with small cell size (arrow head), necessary to accurately calculate ESS. Towards the inner lumen of the vessel, cell size increases to reduce the total number of cells and thus computation time.

The axial CTA data sets were digitally processed to extract the geometric contours of the coronary arteries. A software prototype (Siemens AG, Healthcare Sector, Forchheim, Germany) was used for semiautomatic segmentation and meshing of the vessel surface with triangles. This model was imported into an open-source software package (MeshLab V1.11). Structures other than the examined vessel were removed. Sidebranches were cut at a distance at least two vessel diameters apart from the bifurcation. The resulting surface mesh model was further processed in a commercially available software tool (Gambit 2.4.6, Ansys Inc., Southpointe, PA, USA). In a first step the previous mesh was removed and replaced by a triangular surface mesh with smaller element size. Inflow and outflow areas were defined. Subsequently an adaptive 3D computational mesh was built consisting of 300,000 to 400,000 polyhedral elements for the entire model. One model per vessel was reconstructed. This model was further used for blood flow simulations (Fig. 1).

#### Model assumptions and boundary conditions

Blood flow was assumed to be constant with a velocity of 0.17 m/sec at the coronary ostium, 3D, incompressible and laminar, based on the low Reynolds number of about 300 for small vessels [12,13]. Blood was defined as Newtonian fluid with a dynamic viscosity of 3.7 mPas and a density of 1060 kg/m^3^. Vessel walls were defined as solid, stiff and stationary. A no slip condition was used at the boundaries of the vessel wall.

#### Computational fluid dynamics

**Figure 2:**
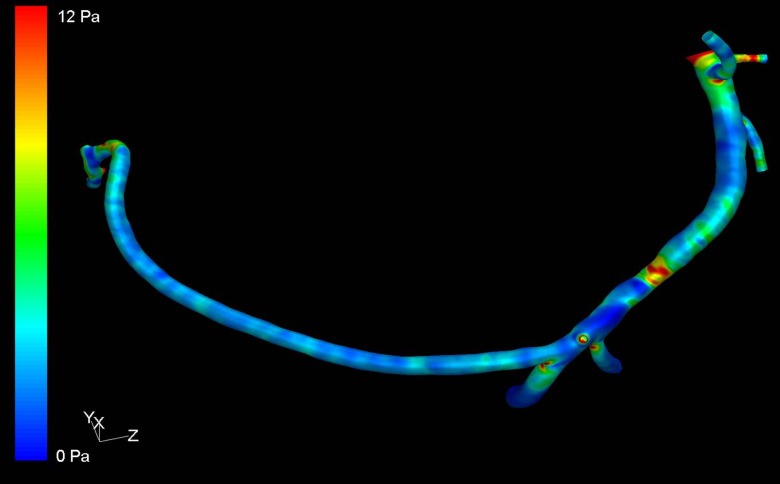
Color encoded illustration of endothelial shear stress (ESS) on a 3D model of a right coronary artery obtained by coronary computed tomography angiography. After segmentation side branches were cut 1–2 cm from the branching point. The volume mesh consisted of about 400,000 polyhedral cells. The Navier-Stokes equations were solved by the finite element method. The level of ESS increases from blue to red as shown in the color map on the left.

**Figure 3:**
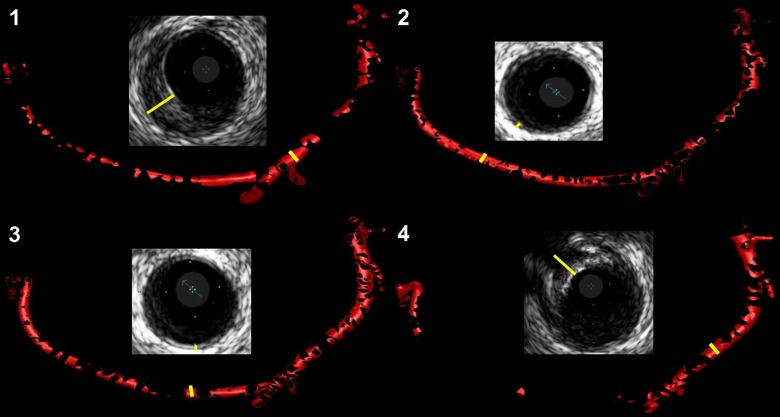
Vessel surface areas corresponding to different levels of endothelial shear stress (ESS). For each quartile of ESS a single model was reconstructed and used for correlation with intravascular ultrasound (IVUS). In quartile 1 (lowest ESS) and quartile 4 (highest ESS) a high prevalence of atherosclerotic plaque was observed whereas intermediate ESS (quartile 2 and 3) was mostly associated with no significant wall changes. The marked sites in the IVUS pictures correspond to marked areas on the surface models.

The Navier-Stokes equations for the individual elements were solved using the finite element method (Fluent 12.0.16, Ansys In., Southpointe, PA, USA). Calculations were performed until the solution fully converged. The results were displayed as a 3D model of the vessel with color coded ESS (Fig. 2). The elements on the surface mesh were divided into quartiles corresponding to different levels of ESS and representing 25% of the mesh surface area (quartile 1 = lowest ESS, quartile 2 = intermediate low ESS, quartile 3 = intermediate high ESS, quartile 4 = highest ESS). Models of the vessel surface for each quartile were reconstructed and exported for analysis (Fig. 3).

#### CTA and IVUS matching

The position of the IVUS probe was determined at the beginning and the end of the motorized pullback by fluoroscopy. Anatomic landmarks which could be detected by both CTA and IVUS were identified, including side branches, the left main coronary artery and atherosclerotic lesions with characteristic features such as calcifications, pronounced eccentric wall thickening or luminal narrowing. Based on the known pullback speed this information was used for manual correlation of the IVUS images and the 3D coronary model obtained by CTA.

#### IVUS measurements

The thickness of the intima-media complex as defined by the lumen border and the signal intense outer elastic lamina was measured in grayscale IVUS images using a dedicated software package (Medical Imaging Assistant, Version 4.2.10C, INDEC BioSystems Inc., Santa Clara, CA, USA). In each ESS quartile, measurements of intima-media thickness were obtained at 25 randomly selected points, resulting in 100 measurements per vessel. The measurements were performed by two experienced investigators who were blinded to the CFD results to avoid bias. To include early atherosclerotic changes the presence of plaque was assumed if the thickness of intima-media complex exceeded 0.3 mm (Fig. 3) [14].

### RF data plaque analysis

**Figure 4:**
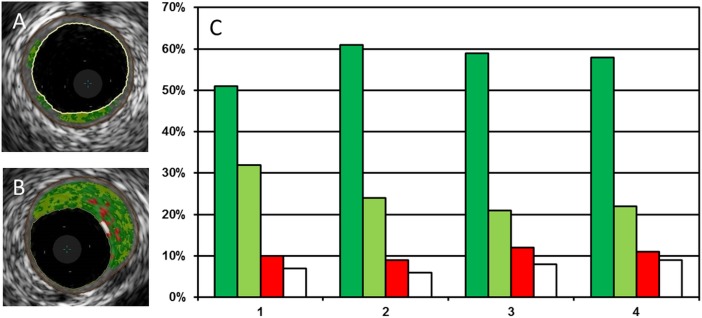
Distribution of plaque tissue composition in areas exposed to different levels of endothelial shear stress (ESS). Panel (A) shows an example of an early, panel (B) an example of a more advanced atherosclerotic lesion as assessed by intravascular ultrasound radiofrequency data analysis. Fibrous tissue is represented by dark green, fibrofatty tissue by light green, necrotic tissue by red and calcified tissue by white colour. We observed a significantly higher amount of fibrofatty (*) tissue in areas exposed to the lowest level of ESS (quartile 1) in comparison to low-intermediate ESS (quartile 2), intermediate-high ESS (quartile 3) or high ESS (quartile 4) (p≤0.023) (C). There was no difference in the amount of other tissue types depending on the level of ESS (p≥0.061).

Data on plaque composition were derived from IVUS RF data analysis (Virtual Histology, VH) using the S5i system (Volcano Corp., Rancho Cordova, CA, USA). For vessel wall definition the lumen-vessel border as well as external elastic membrane were automatically detected and manually corrected in each selected cross-section. For each cross-section the relative amount of fibrofatty tissue, fibrous tissue, necrotic core and dense calcium is calculated by the software (Fig. 4) [15].

### Statistical analysis

All numerical data are presented as mean ± standard deviation. A chi-square test was used for the comparison of plaque presence in the different quartiles. For the comparison of wall thickness and plaque composition an analysis of variance and post hoc Duncan’s multiple range tests were performed. To account for the clustered nature of the data a general estimation equation was applied. The level of significance was set at p<0.05. A commercially available software tool (SPSS Statistics 17.0, SPSS Inc., Chicago, IL, USA) was used for statistical analysis.

## Results

### Patients

Of the 14 patients who underwent coronary CTA, 7 (50%) had an indication for cardiac catheterization based on CTA findings and current guidelines [16]. IVUS was attempted in 18 of 21 (86%) vessels and was successfully performed in 14 of 21 (67%) arteries. The quality of coronary CTA was sufficient in 18 of 21 (86%) vessels but only 12 of 21 (57%) could be successfully segmented and meshed. In the remaining 6 of 21 (29%) arteries calcifications (3 vessels), strong enhancement of an adjacent coronary vein (1 vessel) or weak enhancement of major side branches (2 vessels) precluded adequate segmentation of the vessel lumen. Based on quantitative coronary angiography, no stenosis with > 30% luminal obstruction was detected. Hence a total number of 10 vessels (1 right coronary artery, 5 left anterior descending arteries and 4 left circumflex arteries) from 7 patients (4 male) with matching coronary CTA and IVUS examinations were available for analysis. Patient characteristics are shown in Table 1.

**Table 1:** Patient characteristics.

Age (years) (mean±SD)	57.1±7.2
Gender (male) (n, %)	4 (57.1%)
Risk factors:	
Diabetes mellitus (n, %)	0 (0%)
Hypertension (n, %)	5 (71.4%)
Smoking (n, %)	3 (42.9%)
Hyperlipidemia (n, %)	3 (42.9%)
Positive family history (n, %)	4 (57.1%)
Medication:	
ASA (n, %)	2 (28.6%)
Beta blockers (n, %)	4 (57.1%)
Calcium channel blockers (n, %)	1 (14.3%)
Statins (n, %)	3 (42.9%)
ACE inhibitors (n, %)	4 (57.1%)
Coronary artery investigated:	
Right coronary artery	1 (10.0%)
Left anterior descending artery	4 (40.0%)
Left circumflex artery	5 (50.0%)

Data based on 7 patients included in the final analysis.

ACE: angiotensin converting enzyme, ASA: acetylsalicylic acid

### Endothelial shear stress

Based on the previously defined conditions the mean ESS in the 10 vessels was 1.66 ± 0.84 Pa (range of 0.02–13.75 Pa). The highest levels of ESS were observed at the inner side of proximal bifurcations whereas the lowest ESS occurred at the inner curvature of angulated segments. There was no significant difference in the average ESS between proximal and distal vessel segments (1.70 ± 1.51 Pa, 95% CI 1.49–1.86 Pa vs. 1.62 ± 0.53, 95% CI 1.43–1.79 Pa, p = 0.18). However, there was a wider distribution of ESS values in the proximal than in the distal parts of the vessel (range 0.02–13.75 Pa vs. 0.11–6.67 Pa). Since the inflow velocity was set as a constant, there was no significant relation between the mean value of ESS and the respective vessel diameter observed. For all vessels the average ESS in quartile 1 was 0.73 ± 0.30 Pa (range 0.02–1.08 Pa), in quartile 2 1.44 ± 0.71 Pa (range 1.09–1.67 Pa), in quartile 3 2.13 ± 1.03 Pa (range 1.68–2.75 Pa) and in quartile 4 3.80 ± 2.7 Pa (range 2.76–13.75 Pa).

### Wall thickness

The average wall thickness for all vessels was 0.34 ± 0.28 mm (range 0.07–2.20 mm). Wall thickness was higher in the proximal than in the distal parts of the vessel (0.46 ± 0.32 mm, 95% CI 0.38–0.61 mm vs. 0.28 ± 0.14 mm, 95% CI 0.18–0.32 mm, p < 0.001). In areas where no plaque was present the average wall thickness was 0.21 ± 0.07 mm (range 0.07–0.30 mm, 95% CI 0.20–0.22 mm). In segments where plaque was found the average wall thickness was 0.69 ± 0.39 mm (range 0.31–2.20 mm, 95% CI 0.62–0.75 mm) (p < 0.001).

#### Plaque prevalence and composition

In total plaque was present in 321 (32.1%) of all measurements with very few calcified regions as defined by CTA (6 of 321 (1.8%)). IVUS RF data analysis for plaque composition was successful in 260 of 321 (81.0%) of all plaque cross-sections. In the remaining 61 (19%) cross-sections the software was not able to analyze tissue components due to very small plaque area. In total 56.3±41.7% of plaque tissue consisted of fibrous, 24.7±22.3% of fibro-fatty, 11.9±7.8% of necrotic and 7.1±5.6% of calcified tissue (all p≤0.012).

### Association of ESS and wall thickness

The arterial wall was thickest in areas of low ESS (quartile 1) followed by areas of high ESS (quartile 4) (p < 0.001). In segments exposed to intermediate ESS (quartile 2 and 3) the wall was significantly thinner (p < 0.001). Average wall thickness in the different quartiles and 95% CI are shown in Table 2.

**Table 2:** Wall thickness.

**Quartile**	**Mean Wall Thickness ± SD (mm)**	**Minimum (mm)**	**Maximum (mm)**	**95% Confidence Interval (mm)**
1	0.43 ± 0.34	0.07	2.08	0.39–0.47
2	0.25 ± 0.18	0.01	1.30	0.23–0.27
3	0.28 ± 0.20	0.06	1.73	0.26–0.31
4	0.38 ± 0.32	0.06	2.20	0.32–0.35

Mean wall thickness was highest in quartile 1 (low endothelial shear stress (ESS)) and lowest in quartiles 2 and 3 (intermediate ESS). Vessel wall thickness in quartile 4 (high ESS) was in between. Differences were not significant between quartile 2 and 3 (p = 0.15). All other differences were statistically significant (p < 0.001).

SD: standard deviation

### Association of ESS and plaque prevalence

Most of plaques were found in quartile 1 (124 of 321, 38.6%) followed by quartile 4 (87 of 321, 27.1%). In the second quartile 50 of 321 (15.6%) and in the third quartile 60 of 321 (18.7%) plaques were found. Atherosclerotic plaque prevalence is shown in Table 3. In short there was a significantly higher prevalence of plaques in areas with very low or very high ESS as compared to intermediate levels of ESS (p < 0.001). Also there were more plaques in areas of low than high ESS (p < 0.001).

**Table 3:** Plaque prevalence.

**Quartile**	**No plaque (n, %)**	**Plaque (n, %)**
1	126 (50.4)	124 (49.6)
2	200 (80.0)	50 (20.0)
3	190 (76.0)	60 (24.0)
4	163 (65.2)	87 (34.8)

There was a significantly higher prevalence of atherosclerotic plaques in areas of very low (quartile 1) and very high (quartile 2) endothelial shear stress (ESS) as compared to areas of intermediate ESS (quartile 2 and 3) (p < 0.001). Furthermore plaque prevalence was higher in quartile 1 compared to quartile 4 (p < 0.001). Differences between quartile 2 and 3 were not significant (p = 0.56).

### Association of ESS and plaque composition

We observed a higher relative amount of fibrofatty tissue in regions exposed to the lowest level of ESS (p≤0.023). There was no significant difference in fibrous, necrotic, and calcified tissue among different ESS levels (all p≥0.061) as shown in Fig. 4.

## Discussion

This pilot study shows that CFD calculations based on 3D models of coronary arteries obtained by non-invasive CTA are feasible and can demonstrate an association between ESS distribution and atherosclerotic plaque pattern in patients without priory known coronary artery disease.

In concordance with earlier experimental series we observed a high number of atherosclerotic lesions, increased wall thickness and higher amount of fibrofatty tissue in areas exposed to low ESS [6,7,17]. The pathophysiologic role of low ESS for the genesis of atherosclerosis has been extensively studied. Low ESS reduces the availability of nitric oxide and increases the production of endothelin leading to endothelial dysfunction [18–20]. Furthermore low ESS fosters the uptake of low-density lipoprotein-cholesterol, activation of apoptotic activity in endothelial cells and upregulation of adhesion molecules for inflammatory cells [21–23]. Increased wall thickness and high amount of fibrofatty tissue are characteristics of these early plaque formations [24,25]. After their initiation, the natural history of early fibroatheromas also depends on the level of ESS. High risk vulnerable plaques tend to occur in areas with the lowest values of ESS [17,26]. In the present study few lesions were found in segments with intermediate ESS. However, we found a high number of atherosclerotic lesions also in areas exposed to high ESS. Although in the past high ESS was considered the driving force for the development of atherosclerosis, it is today confirmed that plaques preferentially develop in regions of low ESS [17,27]. Nevertheless, large atheromas can cause obstruction of the vessel lumen. In segments with even mild luminal narrowing blood flow velocity can increase and ESS rises subsequently [28]. To minimize this effect, vessels with a significant lesion (> 30% by quantitative coronary angiography) have been excluded. We were unable to detect differences in the relative amount of fibrous, necrotic and calcified tissue in areas exposed to low, intermediate or high levels of shear stress. Given the large standard deviations, the present study might be underpowered to detect these differences. This is especially true for the rather small amounts of calcifications and necrotic tissue that we found in this low risk population, which are typical features of advanced plaque types [29].

The importance of low ESS has recently been underscored by the PREDICTION study. In patients suffering from acute coronary syndrome low ESS was an independent predictor for progressive plaque enlargement and luminal narrowing despite good medical therapy for risk factor reduction [5]. This and other studies used techniques for ESS assessment that depend on invasive procedures like conventional angiography and IVUS, which are adequate in a high risk population [5,6,28]. However, invasive procedures are not adequate in low risk populations. Therefore these patients often undergo coronary CTA as part of the work-up for suspected coronary artery disease.

Data on ESS analysis based on non-invasive coronary CTA models is currently limited [8,9]. A recent study by Katranas et al. demonstrated an association of low ESS and expansive plaque remodeling [30]. In our study we found a relationship between ESS and plaque prevalence, wall thickness as well as plaque composition adding to the body of evidence for the feasibility of CTA derived ESS analysis. Furthermore our study confirms the association of low ESS and atherosclerotic plaque in a low to intermediate risk population without a history of coronary artery disease. Invasive angiography and IVUS examinations were not used for CFD analysis but were only performed for accurate plaque assessment, which allowed evaluation of even very early plaque stages. One major drawback of ESS assessment with coronary CTA without additional invasive procedures is its limited spatial resolution which would prevent detection of subtle atherosclerotic changes. However, in a natural-history study of atherosclerosis most lesions that caused subsequent cardiovascular events had a high plaque burden of about 70% and a small luminal area [31], features which can also be appreciated in coronary CTA [32,33]. If large, soft plaques are detected on coronary CTA, ESS analysis might allow for a better prediction of progression or subsequent events, a thesis which needs to be tested in further trials. Furthermore the use of CTA might facilitate the analysis of the relationship of high risk plaque features and ESS in large patient cohorts.

The absolute values for ESS were comparable to other studies [6,7,13,17]. Nevertheless absolute values of ESS highly depend on the lumen of the 3D vessel reconstruction and the boundary conditions of the CFD model. Automated or semi-automated vessel segmentation may affect the absolute dimensions of the vessel. Lumen area and diameter may be increased or decreased by segmentation procedures subsequently leading to lower or higher absolute numbers of ESS. A higher in-flow velocity in the CFD model results in higher absolute ESS values, whereas the pattern of ESS distribution remains similar over a broad range of inflow velocities. Therefore we did not use absolute but relative ESS values to analyze the association between ESS and atherosclerosis.

This study only shows the correlation between ESS and atherosclerotic plaque at one point in time. Therefore we cannot prove that ESS as calculated by our approach is the driving force behind the atherosclerotic wall changes, as suggested in other studies [5,6]. Serial examinations are necessary to address this question.

### Limitations

Several limitations apply to this study. The study size was rather small with a total number of 7 patients and 10 vessels. Calcifications were the most important cause for inaccurate vessel segmentation precluding adequate CFD analysis in three arteries with diagnostic image quality. Advanced segmentation algorithms will have to be implemented to reduce artefacts caused by calcified plaque and to allow CTA based CFD analysis in patient with extensive calcifications. Since this is an in-vivo study, we were unable to obtain histology data for the analysis of plaque composition but had to rely on IVUS RF data. For the flow simulations we made several simplifications. First of all, blood flow is not constant but changes during the cardiac cycle. Second, we assumed stiff, none moving vessel walls although the vessel is expanding and contracting and is bent while the heart is beating. It can be speculated that a more sophisticated model would find a closer correlation between ESS and atherosclerotic lesions. However, the same simplifications have been made in other studies by different workgroups [5,34,35]. Furthermore, calculations using pulsatile blood flow and deformable meshes require higher computational power and are more time consuming, which might hamper the practical implementation of this technique. Results of ESS calculations based on CTA were not validated against invasive ESS measurements. However, we are not aware of an approved system that allows a direct measurement of ESS in patients in-vivo. The correlation between IVUS and CTA was done manually which probably resulted in some inaccuracy, especially with regard to the rotational orientation of the IVUS images. Co-registration of IVUS and angiography and automated correlation with CTA could help to facilitate this procedure. Moreover, we did not use a patient specific flow profile but a constant, standardized flow, which could result in some inaccuracies for the ESS values compared to the real situation in-vivo. Nevertheless relative ESS quartiles were used to assess the association of ESS and atherosclerosis pattern to reduce the effect of absolute differences in ESS calculations.

## Conclusion

Coronary CTA is an established tool for the non-invasive assessment of coronary morphology. As we were able to demonstrate, CFD analysis based on coronary CTA offers the possibility to gather also information about coronary physiology and functional data non-invasively within one examination. Further studies will evaluate whether ESS analysis based on CTA models can add significant information about plaque composition, progression and risk prediction, which might influence therapeutic decisions. Since coronary CTA is already available in most cardiac centers the potential future implementation of CFD would not depend on new costly hardware development but could be achieved by adding dedicated software packages to common CT-scanners.
